# Microstructure Characterization of Bone Metastases from Prostate Cancer with Diffusion MRI: Preliminary Findings

**DOI:** 10.3389/fonc.2018.00026

**Published:** 2018-02-16

**Authors:** Colleen Bailey, David J. Collins, Nina Tunariu, Matthew R. Orton, Veronica A. Morgan, Thorsten Feiweier, David J. Hawkes, Martin O. Leach, Daniel C. Alexander, Eleftheria Panagiotaki

**Affiliations:** ^1^Centre for Medical Image Computing, University College London, London, United Kingdom; ^2^Department of Radiation Oncology, Odette Cancer Centre, Toronto, Canada; ^3^CR-UK and EPSRC Cancer Imaging Centre, Institute of Cancer Research, Royal Marsden NHS Foundation Trust, London, United Kingdom; ^4^Radiology, Royal Marsden NHS Foundation Trust, Institute of Cancer Research, Sutton, United Kingdom; ^5^Siemens Healthcare GmbH, Erlangen, Germany; ^6^Clinical Imaging Research Centre, National University of Singapore, Singapore, Singapore

**Keywords:** diffusion MRI, bone metastases, kurtosis, intravoxel incoherent motion, vascular, extracellular, and restricted diffusion for cytometry in tumors

## Abstract

**Purpose:**

To examine the usefulness of rich diffusion protocols with high *b*-values and varying diffusion time for probing microstructure in bone metastases. Analysis techniques including biophysical and mathematical models were compared with the clinical apparent diffusion coefficient (ADC).

**Methods:**

Four patients were scanned using 13 *b*-values up to 3,000 s/mm^2^ and diffusion times ranging 18–52 ms. Data were fitted to mono-exponential ADC, intravoxel incoherent motion (IVIM), Kurtosis and Vascular, extracellular, and restricted diffusion for cytometry in tumors (VERDICT) models. Parameters from the models were compared using correlation plots.

**Results:**

ADC and IVIM did not fit the data well, failing to capture the signal at high *b*-values. The Kurtosis model best explained the data in many voxels, but in voxels exhibiting a more time-dependent signal, the VERDICT model explained the data best. The ADC correlated significantly (*p* < 0.004) with the intracellular diffusion coefficient (*r* = 0.48), intracellular volume fraction (*r* = −0.21), and perfusion fraction (*r* = 0.46) parameters from VERDICT, suggesting that these factors all contribute to ADC contrast. The mean kurtosis correlated with the intracellular volume fraction parameter (*r* = 0.26) from VERDICT, consistent with the hypothesis that kurtosis relates to cellularity, but also correlated weakly with the intracellular diffusion coefficient (*r* = 0.18) and cell radius (*r* = 0.16) parameters, suggesting that it may be difficult to attribute physical meaning to kurtosis.

**Conclusion:**

Both Kurtosis and VERDICT explained the diffusion signal better than ADC and IVIM, primarily due to poor fitting at high *b*-values in the latter two models. The Kurtosis and VERDICT models captured information at high *b* using parameters (Kurtosis or intracellular volume fraction and radius) that do not have a simple relationship with ADC and that may provide additional microstructural information in bone metastases.

## Introduction

Bone metastases, the most common site of metastatic disease in prostate cancer, are associated with reduced quality of life and an increased risk of complications from bone weakness. Histology is not generally feasible in bone and there are currently no imaging criteria for detecting benefit or improvement following therapy in osteoblastic bone metastases (1). There is, therefore, a need for new imaging techniques and those sensitive to the tissue microstructure, such as the apparent diffusion coefficient (ADC), have shown promise in predicting response (2).

However, ADC is a relatively broad reflection of the tissue microstructure. Studies in other tumor types demonstrate that more advanced diffusion techniques can provide additional information. For example, the use of more extensive imaging protocols, including high *b*-values, results in higher diagnostic accuracy in prostate cancer (3). Also, the inclusion of varying diffusion times reveals information about important length scales, such as the cell size and organization, as has been demonstrated in breast cancer (4).

With more extensive imaging protocols, it is possible to fit more complex models. For example, the intravoxel incoherent motion (IVIM) model uses low *b*-value data to provide information about vascular perfusion in addition to tissue diffusion (5, 6). The Kurtosis method captures some of the non-Gaussian diffusion effects at higher *b*-values. These may be caused by restricted diffusion such as that inside cells, but the Kurtosis model does not make biophysical assumptions about the source of non-Guassian diffusion (7). In contrast, Vascular, extracellular, and restricted diffusion for cytometry in tumors (VERDICT) is an adaptable framework (8) that uses a biophysical model to describe the tissue in terms of vascular, extracellular, and intracellular features. The vascular compartment is described by a pseudo-diffusion coefficient and a separate extracellular diffusion compartment, similar to the two components in IVIM, although VERDICT allows alternative compartment shapes to be considered. Diffusion in the intracellular space is modeled by a restricted compartment that captures non-Gaussian diffusion effects at high *b*-values.

Although extended diffusion protocols and more complex fitting models have proven useful in revealing non-invasive microstructure indices in breast (9) and prostate (10), they have not yet been examined in bone metastases. This study explores the potential of microstructure characterization in bone metastases using a rich diffusion protocol with *b*-values up to 3,000 s/mm^2^ and a range of diffusion times, then fitting with a variety of diffusion models.

## Materials and Methods

### Patient Cohort

Eight regions of interest (ROIs) were identified by a radiologist (Nina Tunariu, 8 years experience) as bone metastases in four patients with prostate cancer. This study was carried out in accordance with the recommendations of Health Insurance Portability and Accountability Act guidelines and the local Research Ethics Committee with written informed consent from all subjects. All subjects gave written informed consent in accordance with the Declaration of Helsinki. The protocol was approved by the local Research Ethics Committee (Royal Marsden NHS Foundation Trust). One ROI contained a significant proportion of normal fatty bone marrow as result of heterogeneous response to therapy.

### Data Acquisition

Data were collected at 1.5 T (MAGNETOM Avanto, Siemens Healthcare, Erlangen, Germany) using a 24-channel spine matrix coil and 12-channel body matrix receive coil. Thirteen fat-saturated axial diffusion-weighted images (DWI) were acquired using a prototype sequence (single-shot 2D pulsed-gradient spin-echo echoplanar acquisition, 2.97 mm × 2.97 mm resolution, 38 cm × 38 cm field of view, 5 mm slice thickness, repetition time TR = 3 s, 3 signal averages). Each scan had a different *b*-value with a corresponding *b* = 0 image; the diffusion scan parameters and echo times (TEs) are listed in Table 1. Gradients were applied in a single direction aligned with the main B_0_ field (0, 0, 1).

**Table 1:** Diffusion scan parameters and echo times.

Gradient duration δ (ms)	Gradient separation Δ (ms)	Effective diffusion time Δ_eff_ = (Δ − δ/3) (ms)	Gradient strength (mT/m)	*b-*Value (s/mm^2^)	TE (ms)
24.8	33.8	25.5	29.8	1,000	77.2
33.2	45.6	34.5	27.1	2,000	94
40	52.4	39.1	25.9	3,000	111.6
40.2	52.6	39.2	23.5	2,500	108
28.2	40.6	31.2	29.1	1,500	84
19	31.4	25.1	35.1	800	67.6
13.4	26.2	21.7	37.1	400	56.4
10	22.8	19.4	37.9	200	49.6
7.4	20.2	17.7	37.9	100	44.4
24.8	60	51.7	14.8	500	100.4
24.8	60	51.7	18.7	800	100.4
24.8	50	41.7	20.9	800	90
24.8	50	41.7	16.5	500	90

### Data Fitting

Data were fitted voxelwise with six different models, outlined in Table 2, using a maximum likelihood procedure that accounts for Rician noise. Noise was estimated by applying the difference method (11) to repeated *b* = 0 measurements in the target ROI.

**Table 2:** Models tested with fit parameters in parentheses.

Model	Vascular	Extracellular	Intracellular	No. parameters^a^
Apparent diffusion coefficient (ADC)	Ball (ADC)			3
Intravoxel incoherent motion	Ball (*f*_p_, *D*_p_)	Ball (*f*_t_, *D*_t_)		4
Kurtosis	Kurtosis (*D*_kurt_, *K*)			4
Astrosticks–Ball–Sphere	Astrosticks (*f*_p_, *D*_p_)	Ball (*f*_E_, *D*_E_)	Sphere (*f*_I_, *D*_I_, *R*)	6
Ball–Ball–Sphere	Ball (*f*_p_, *D*_p_)	Ball (*f*_E_, *D*_E_)	Sphere (*f*_I_, *D*_I_, *R*)	6
Ball–Astrosticks–Sphere	Ball (*f*_p_, *D*_p_)	Astrosticks (*f*_E_, *D*_E_)	Sphere (*f*_I_, *D*_I_, *R*)	6

*Total number of fit parameters summarized in the last column*.

*^a^Number of model parameters includes normalization constant (S_0_) and T2 decay time constant (T2). Additional constraints: D_p_ = 1 × 10^−2^ mm^2^/s, D_E_ = D_I_ < 3 × 10^−3^ mm^2^/s, ∑f_j_ = 1*.

Signal equations for the models were calculated as the sum of the individual compartmental diffusion signals, *S_j_*, weighted by their volume fraction, *f*_j_:

$$S=S_{0}e^{-\text{TE/T2}}\sum_{j}f_{\text{j}}S_{\text{j}},$$

where TE is the echo time. All models included a signal normalization constant (S_0_) and a T2 time constant (T2). These parameters were estimated from the global fit to all data to account for differing echo times. A separate T2 fit of all *b* = 0 points confirmed that T2 decay was mono-exponential. The signal equations for the compartments, *S*_j_, are as follows; derivations are found in Ref. (7, 12).

Kurtosis (7) is a phenomenological model that attempts to describe deviations from Gaussian diffusion using the apparent diffusion kurtosis, *K*:

$$S_{\text{Kurt}}=e^{-bD_{\text{kurt}}+b^{2}D_{\text{kurt}}^{2}K/6},$$

with $b={\left(\gamma g\delta \right)}^{2}(\Delta -\delta /3)$, where γ is the gyromagnetic ratio, g is the gradient strength, δ is the gradient duration, and Δ is the gradient separation.

The Ball compartment describes Gaussian diffusion:

$$S_{\text{Ball}}=e^{-bD_{\text{ball}}}.$$

The Astrosticks compartment assumes a uniform distribution of Sticks whose diffusion is restricted to a single dimension:

$$S_{\text{Astrosticks}}=\pi^{1/2}{\left(2\sqrt{bD_{\text{Stick}}}\right)}^{-1}\phi \left(\sqrt{bD_{\text{Stick}}}\right),$$

where $\phi (z)=2\pi^{-1/2}\int_{0}^{z}\text{exp}(-t^{2}\text{​})\;$ is the error function.

The Sphere compartment calculation uses the GPD approximation (13):

$$S_{\text{Sphere}}=\exp\left(-2\gamma^{2}g^{2}\sum_{\text{m}}\frac{{2\beta }_{\text{m}}{\;}^{2}D_{1}\delta -2+2Y\left(\delta \right)+2Y\left(\Delta \right)-Y\left(\Delta -\delta \right)-Y\left(\Delta +\delta \right)}{D_{1}{\;}^{2}\beta_{\text{m}}{\;}^{6}\left(\beta_{\text{m}}{\;}^{2}R^{2}-2\right)}\right).$$

Here, $Y(x)=e^{-{\beta_{\text{m}}}^{2}D_{1}x}$, β_m_ is the mth root of $J_{3/2}(\beta_{\text{m}}R)-\beta_{\text{m}}R\;J_{5/2}(\beta_{\text{m}}R)=0$ and J_ν_ is the Bessel function of the first kind, order ν. The summation was carried out over the first 31 roots of the equation.

In this nomenclature, the ADC is a parameter in the single-compartment Ball fit. The IVIM model is the sum of two Ball compartments, one representing vasculature with pseudodiffusion coefficient *D*_p_ and fraction *f*_p_ and a second representing tissue with diffusion coefficient *D*_t_. VERDICT models are constructed as the sum of signals from vascular pseudodiffusion (p), extracellular (E), and restricted intracellular sphere (I) compartments with fractions *f*_p_, *f*_E_, and *f*_I_ and diffusion coefficients *D*_p_, *D*_E_, and *D*_I_, respectively, with *D*_E_ = *D*_I_ assumed. The average radius of the spherical intracellular compartment was modeled by the parameter *R*. Different compartment shapes were tested for the vascular and extracellular spaces in VERDICT, and these models are named in vascular–extracellular–intracellular order using the terminology in Ref. (12): Astrosticks–Ball–Sphere (ABS), Ball–Ball–Sphere (BBS), and Ball–Astrosticks–Sphere (BAS).

The pseudodiffusion coefficient was found to have little influence on the fit and was fixed to *D*_p_ = 1 × 10^−2^ mm^2^/s and the intra- and extracellular diffusion coefficients were assumed to be equal and less than 3 × 10^−3^ mm^2^/s. Volume fractions summed to one. Sphere radius *R* was constrained between 0.1 and 20 μm.

### Statistical Analysis

Models were compared using the Akaike Information Criterion (AIC) (14), where lower AICs indicate models that explain the data better after accounting for varying numbers of model parameters. Graphs present the relative difference in AIC: ΔAIC=AIC–AIC_min_, where AIC_min_ is the lowest AIC of all six models in each voxel.

The relationship between model parameters was examined using correlation plots. Linear regression was performed on all voxels from the seven non-fatty ROIs pooled together and the Pearson correlation coefficient was calculated. Correlations were considered significant if *p* < 0.004 (*p* < 0.05/12, to account for the 12 pairs of variables compared).

## Results

**Figure 1:**
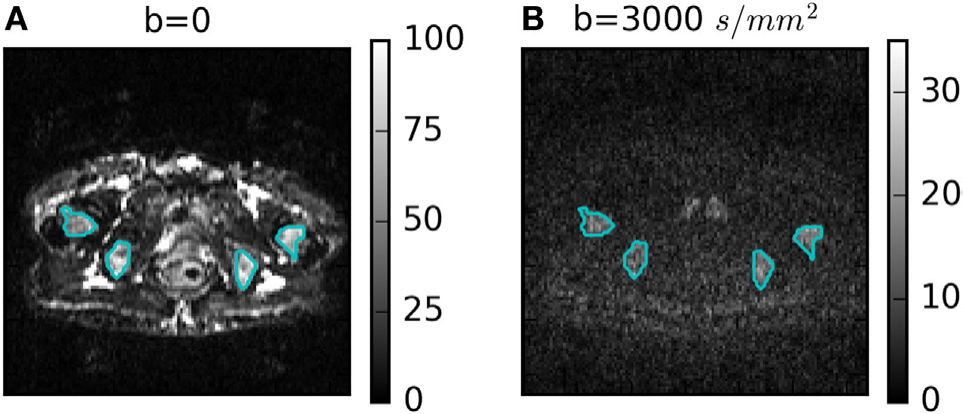
**(A)** *b* = 0 and **(B)** *b* = 3,000 s/mm^2^ diffusion-weighted images for one patient with four regions of interest outlined in cyan.

Figure 1 shows a representative DWI for *b* = 3,000 s/mm^2^ with its corresponding *b* = 0 image. Four ROIs defining bone metastases are outlined in cyan. Heterogeneity is visible in the *b* = 0 and *b* = 3,000 s/mm^2^ images, but with different patterns. The lowest average SNR in an ROI at *b* = 3,000 s/mm^2^ was 10.

**Figure 2:**
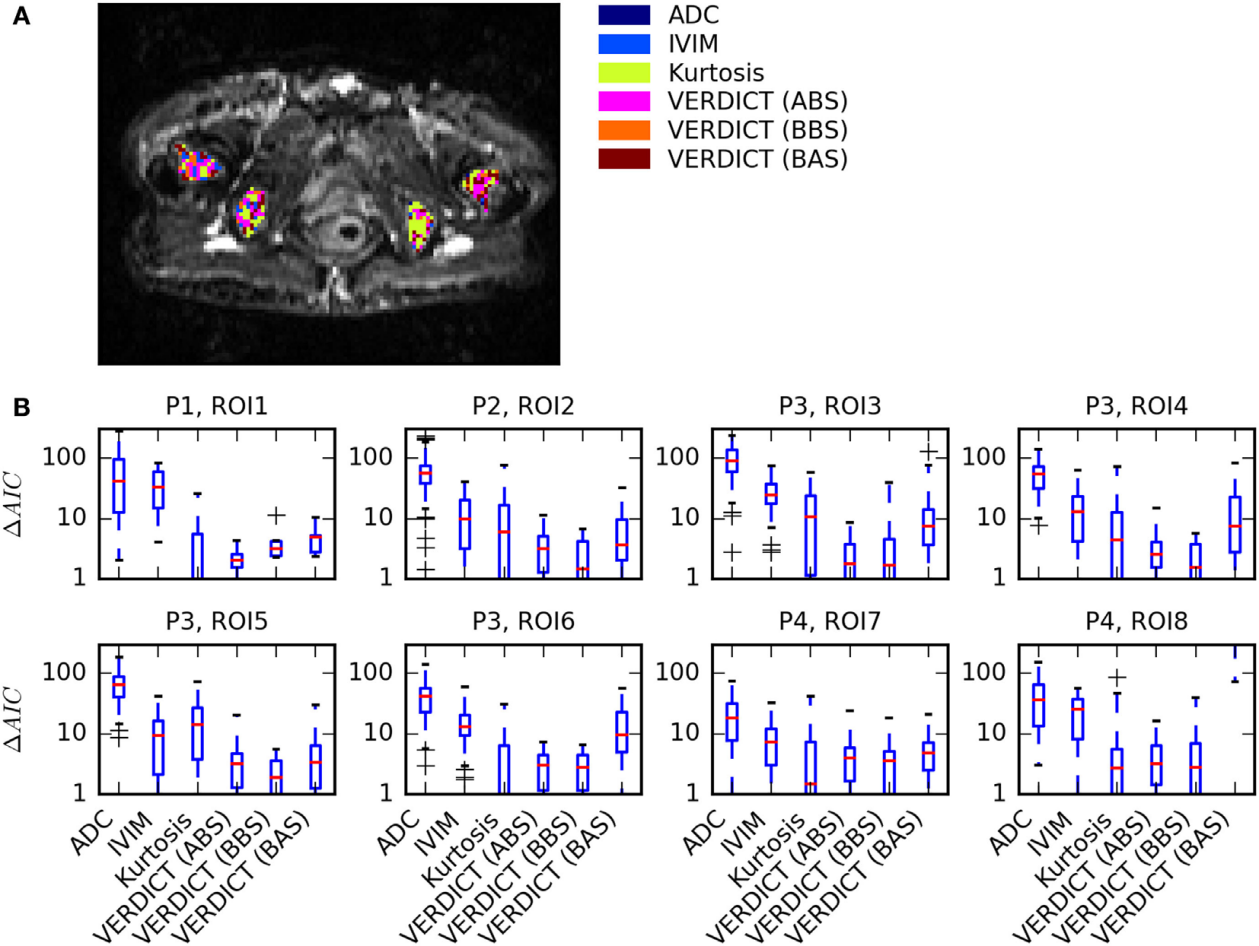
Summary of model selection results. **(A)** Map showing the model that best explained the data [lowest Akaike Information Criterion (AIC)] in each voxel for one patient. **(B)** Boxplots of ΔAIC, where the red median lines indicate that Kurtosis, Astrosticks–Ball–Sphere (ABS), and Ball–Ball–Sphere (BBS) models tend to have similarly low AICs in most voxels, but the larger upper range of the Kurtosis box indicates that some voxels are better explained using VERDICT.

Figure 2 summarizes the model selection results. Figure 2A presents a map of the model that best explained the data in each voxel in four ROIs of one patient. There were very few voxels where ADC and IVIM models best explained the data. Many voxels were sufficiently explained by the Kurtosis model, but a small number of voxels were better explained by the more complex VERDICT models. This was consistent across all ROIs, as shown by the boxplots in Figure 2B, which demonstrate that ADC and IVIM models have much higher median ΔAIC across all voxels. Kurtosis and two of the VERDICT models (ABS and BBS) both tend to have low median ΔAIC, although the interquartile range of the Kurtosis ΔAIC is higher because some voxels were very poorly explained by Kurtosis. The BAS VERDICT model performed consistently worse than the other VERDICT models. Since the ABS and BBS VERDICT models yielded similar fits and parameters, results for BBS are presented in subsequent figures, but ABS results were similar.

**Figure 3:**
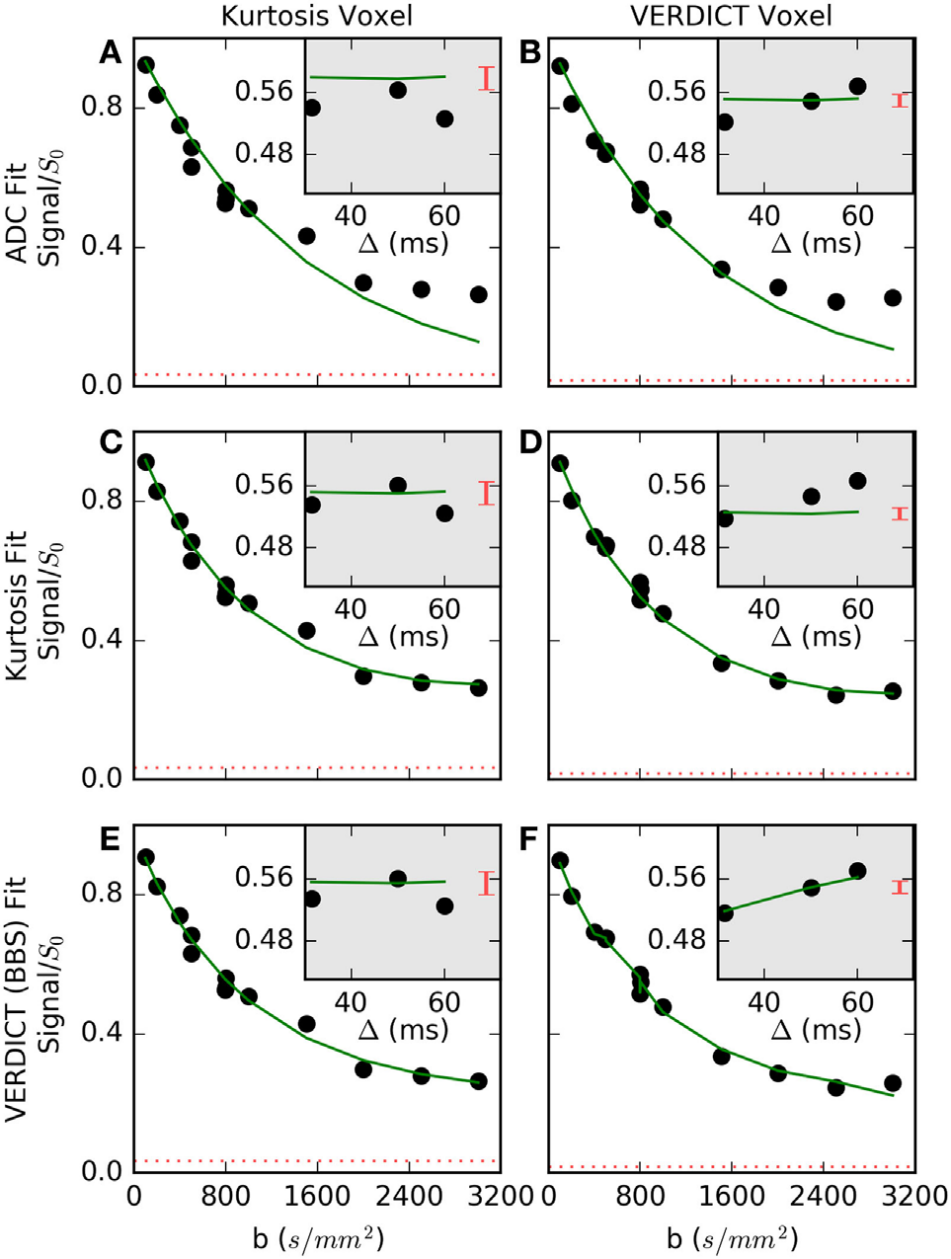
Normalized signal and selected fits are shown for **(A,C,E)** a voxel where Kurtosis best described the data and **(B,D,F)** a voxel where Vascular, extracellular, and restricted diffusion for cytometry in tumors (VERDICT) [Ball–Ball–Sphere (BBS)] best described the data. The apparent diffusion coefficient (ADC) model **(A,B)** failed at high *b*-values in both voxels. In the first voxel **(C,E)**, the Kurtosis and VERDICT models provided similar fits, so, Kurtosis was selected as the simpler model. In the second voxel **(D,F)**, the gray insets showed the signal for three points with *b* = 800 s/mm^2^, where Kurtosis is unable to capture signal changes with different gradient separations, Δ, and VERDICT better explained this time-dependence. The dashed red lines and the red bars in the insets both indicate the noise level, demonstrating that it is difficult to detect a time-dependent trend in the first voxel beyond the larger relative noise level.

Figure 3 shows the fit in more detail for two voxels (columns) that are representative of (a, c, e) voxels where Kurtosis best explained the data and (b, d, f) voxels where VERDICT best explained the data. The simple ADC model (a, b) failed at high *b*-values in both voxels. The Kurtosis model (c, d) is better able to capture non-Gaussian behavior at high *b*. Where Kurtosis best explained the data, the VERDICT fit was similar (c versus e), but Kurtosis was selected as the simpler model. The gray insets separate out three data points with *b* = 800 s/mm^2^, but with different gradient separations and diffusion times. Where VERDICT best explained the data (d versus f), there was greater diffusion time-dependence relative to the noise (indicated by the red bars). The diffusion time-dependence at constant *b*-value cannot be explained using the Kurtosis model (flat green line in c, d insets), but is captured by the restricted sphere component in VERDICT (f inset).

**Figure 4:**
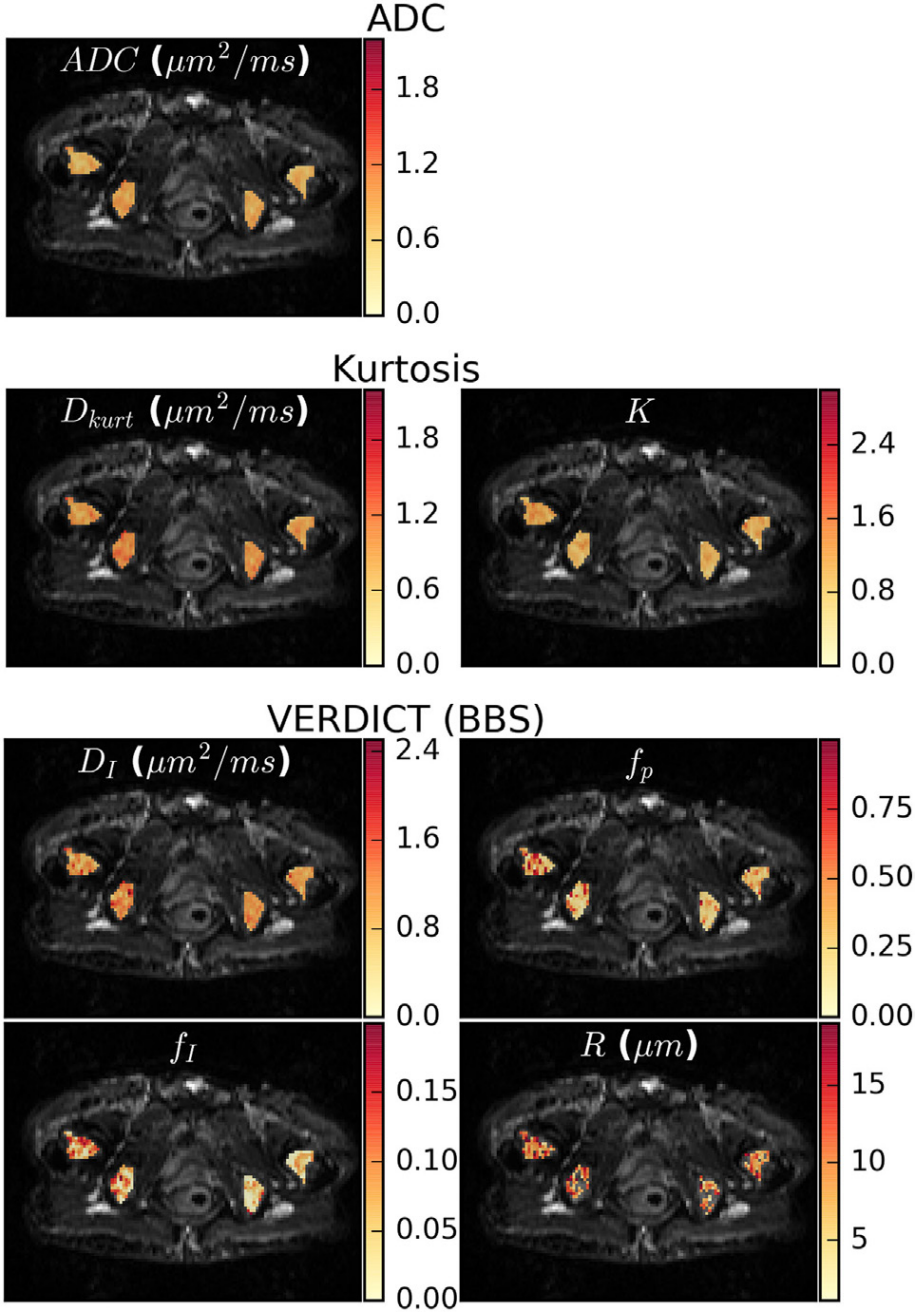
Parameter maps for the apparent diffusion coefficient (ADC), kurtosis, and vascular, extracellular, and restricted diffusion for cytometry in tumors (VERDICT) [Ball–Ball–Sphere (BBS)] models. There was some apparent heterogeneity in the ADC map and the *D*_kurt_ and *D*_I_ parameters from the kurtosis and VERDICT models showed similar patterns. For VERDICT, the intracellular volume fraction (*f*_I_) and perfusion fraction (*f*_p_) maps appeared heterogeneous, but with a different pattern. Several voxels in the radius (*R*) maps hit the fitting lower bound and were not plotted. These correspond to regions where the Kurtosis model provided better fit in Figure 2A and had low f_I_, meaning that the radius was poorly determined. Parameter maps for the remaining patients can be seen in the Supplementary Material.

Figure 4 shows parameter maps in one patient for the ADC, Kurtosis, and VERDICT models (maps for other patients are presented in the Supplementary Material). The diffusion coefficients for all models (ADC, *D*_kurt_, and *D*_I_) appeared heterogeneous. However, the potential heterogeneity in the intracellular and pseudodiffusion maps, *f*_I_ and *f*_p_, respectively, had a different pattern. The radius parameter varied between ~6 and 12 μm, although several voxels (not plotted) reached the lower bound allowed by the fitting; these had low *f*_I_ so that *R* was poorly determined.

**Figure 5:**
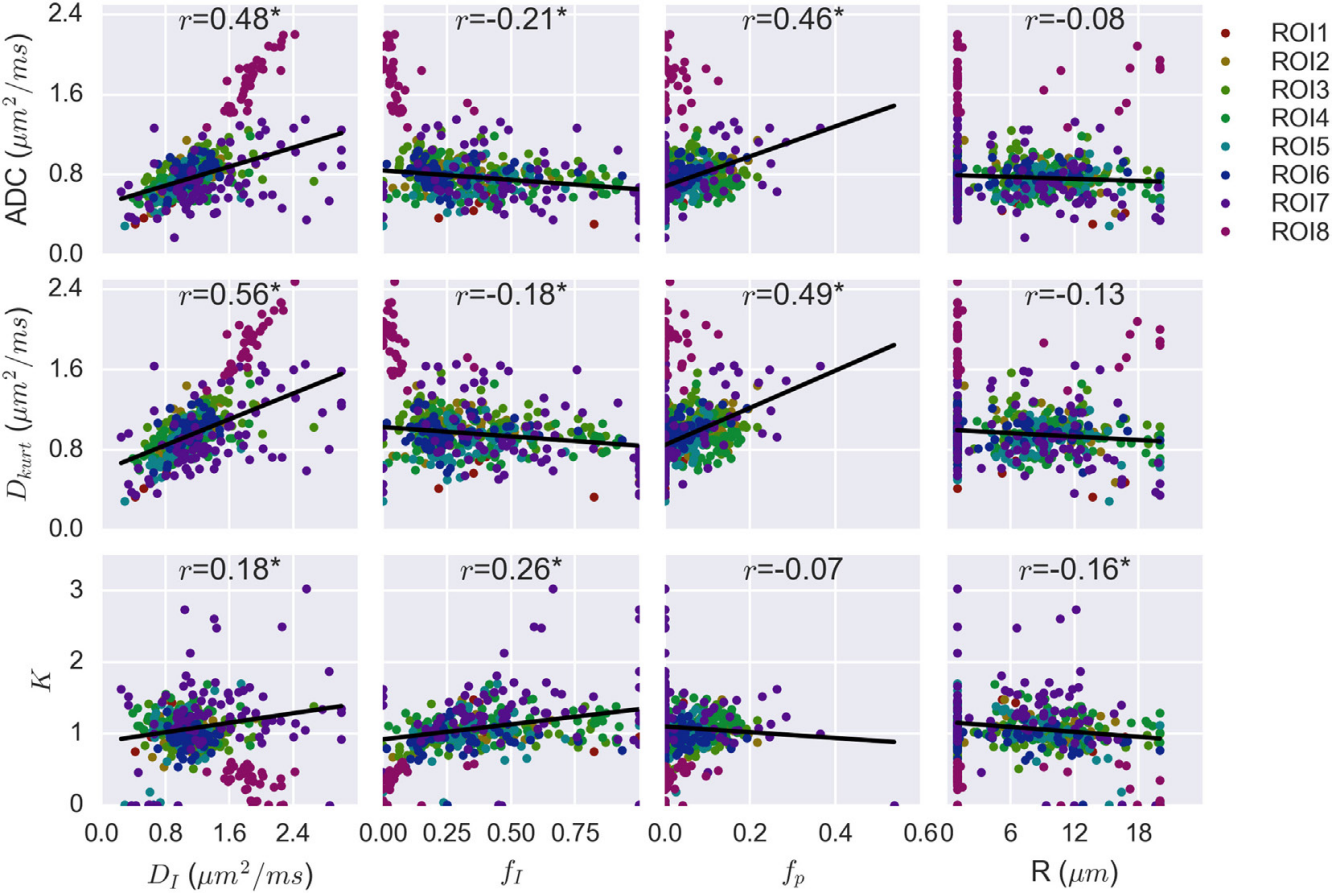
Correlation plots combining voxel data for all patients, with regions of interest (ROIs) separated by color. Linear regression on combined data from the seven non-fatty ROIs demonstrated significant correlation between apparent diffusion coefficient (ADC) and vascular, extracellular, and restricted diffusion for cytometry in tumors (VERDICT) [Ball–Ball–Sphere (BBS)] parameters *D*_I_, *f*_I_, and *f*_p_. These same three VERDICT parameters correlate with *D*_kurt_, whereas *K* correlates with *f*_I_ and, more weakly, with *R* and *D*_I_.

Figure 5 plots the correlation between the VERDICT (BBS) model parameters and those from the ADC and Kurtosis models. The data from the fatty ROI (ROI8) do not align exactly with the trends from other ROIs. The fatty ROI exhibited the highest ADC, *D*_kurt_ and *D*_I_, but had low *f*_I_ and *f*_p_. Low *f*_I_ also resulted in a poorly determined *R* value that defaulted to the lower fitting limit for almost all voxels in this ROI. Due to the deviation from the trends followed by the less fatty voxels and the influence of points at the extreme ends of the parameter space, voxels from the fatty ROI were not included in the linear regression and Pearson correlation calculation.

The Pearson correlation coefficients are shown above each plot and the asterisk indicates a statistically significant correlation (*p* < 0.004). Both ADC and *D*_kurt_ correlated significantly with *D*_I_ (*p* = 7 × 10^−25^, 4 × 10^−35^ for ADC and *D*_kurt_, respectively), *f*_I_ (*p* = 10^−5^, 2 × 10^−4^) and *f*_p_ (*p* = 3 × 10^−23^, 10^−26^). The kurtosis parameter, K, correlated most strongly with the intracellular water fraction, *f*_I_ (*p* = 10^−7^), and more weakly with the diffusion coefficient, *D*_I_ (*p* = 3 × 10^−4^), and radius parameter, *R* (*p* = 10^−3^). *R* did not significantly correlate with the ADC (*p* = 0.09) or *D*_kurt_ (*p* = 0.01).

## Discussion

Although high *b*-values are beginning to be adopted in primary prostate tumors (10, 15) and brain glioma (16), along with models to characterize cellularity and microstructure, these methods had not been studied in bone metastases, where microstructure differs. Microstructure methods in bone would be particularly valuable due to the challenge of acquiring histological information from these areas. This study acquired diffusion data in bone metastases from protocols including high *b*-values and varying diffusion times, demonstrating that these data were not adequately described by conventional ADC and IVIM models. This is consistent with findings from other tumor types (8, 15, 17) and expected in regions with complex and heterogeneous microstructure, including cellular regions.

The kurtosis and VERDICT models captured the signal at high *b*-values and non-Gaussian effects, likely due to intracellular water in these voxels. Kurtosis was often sufficient to characterize the data. However, there were some voxels where the more complex VERDICT model was required to fully describe the signal, particularly variations with diffusion time. These voxels tended to have the largest intracellular volume fraction estimates in parameter maps, which suggest that VERDICT explains the data better than Kurtosis in regions with a larger intracellular signal contribution, resulting in both higher SNR and stronger time-dependence. Kurtosis is not typically performed with varying diffusion time and may be sufficient to describe the data from such protocols.

Of the three VERDICT models tested, BAS did not fit as well as the ABS or BBS models, suggesting that Astrosticks is not a good description of the extracellular space. BBS and ABS demonstrated similar fits and parameter values. These models differ only in the shape of the vascular compartment, and it is likely that low vascular fraction (*f*_p_ < 0.20) makes it difficult to describe the shape accurately.

The median ADC was calculated in each ROI and the mean across ROIs was 0.85 × 10^−3^ mm^2^/s (range 0.43–1.78 × 10^−3^ mm^2^/s; the mean was 0.71 × 10^−3^ mm^2^/s when the fatty ROI was excluded as an outlier at the upper end of the range). This average is within the range reported in previous bone metastasis studies that used a more limited diffusion protocol (2), although the range in this study skews lower, as expected with the inclusion of higher *b*-values. In cellular regions, images at high *b*-values will be less attenuated, resulting in lower ADCs when a simple exponential model is used to fit all data. Previous studies have noted that the relationship between ADC and cell density is complex in bone marrow due to displacement of fat cells as tumor cells infiltrate and the return of fat cells during treatment response (18). Fat cells may have low water content and thus low signal, complicating the ADC fit. The presence of a wider range of *b*-values and careful accounting for noise in this study may explain the high ADC in the fatty ROI. The VERDICT model parameters also support this: the ADC was negatively correlated with the intracellular fraction *f*_I_ and the estimate of *f*_I_ in the fatty ROI was very low, suggesting little intracellular water influenced the diffusion signal in fat-containing voxels.

The VERDICT model parameters fell within plausible biological ranges. The median *D*_I_ of (1.1 ± 0.3) × 10^−3^ mm^2^/s was higher than the ADC because restriction is accounted for and in agreement with previous studies (8). There are challenges to obtaining histology in bone metastases, but the prostate cell size range observed in cell culture of 13 ± 3 μm (19) is in agreement with the BBS VERDICT size estimate (2 × *R* = 15 ± 3 μm). The intracellular fraction parameter *f*_I_ was 0.3 ± 0.1, somewhat lower than observed in primary prostate tumors (10), which may be due to the heterogeneous way in which the metastasis infiltrates the bone and partial volume effects. The low perfusion fraction, *f*_p_ = 0.04 ± 0.03, was low, as expected in bone metastases. The maps also suggested patterns of heterogeneity: ADC, *D*_kurt_, and *D*_I_ appeared similar, with lower values toward the center of the bone, but *f*_p_ and *f*_I_ demonstrate different patterns. The meaning and biological importance of these patterns is of interest for future study.

The correlations were in agreement with previous findings from prostate tumors (10). Both ADC and *D*_kurt_ correlated with *D*_I_, *f*_I_, and *f*_p_, indicating that the parameters from the biophysical VERDICT models all contribute to the values of diffusion coefficients from simpler models and thus these diffusion coefficients are less likely to be specific to microstructural properties. The apparent kurtosis *K* correlated most strongly with *f*_I_, but showed weaker correlation with *R* and *D*_I_. This supports the hypothesis that *K* increases with increasing tissue complexity (20), but also indicates that it may be difficult to separate out different microscopic effects using kurtosis. The radius, *R*, had no correlation with ADC, which is expected given that the effects of restriction are strongest at high *b*-values and the ADC model fits these *b*-values poorly.

There are several limitations to this study. It involves a relatively small number of patients. However, even this small number demonstrates that high *b*-values and varying diffusion times provide information not captured by current clinical methods that may be valuable in measuring tumor response. Future work will explore whether VERDICT or Kurtosis parameters improve prediction of patient outcome in a larger cohort.

The models are all necessarily simplifications of complex biological systems to make data fitting tractable. The T2 was modeled as a mono-exponential decay, an assumption confirmed by fitting all *b* = 0 data. This confirms the efficacy of the fat saturation, which eliminates most signal from aliphatic protons, although the use of longer echo times may also attenuate signal from fat in this study. The number of VERDICT parameters was reduced by assuming the intra- and extracellular diffusion coefficients were equal. It has been demonstrated that the use of short diffusion times or specialized oscillating gradient sequences and high SNR are needed to accurately determine the intracellular diffusion coefficient (21). This is not generally feasible with a clinical protocol, necessitating an assumption about the intracellular diffusion and the equality assumption has been successfully applied in other work (10). Alternative assumptions, such as a tortuosity approximation for the extracellular diffusion coefficient, are also possible. Incorrect assumptions about intracellular diffusion would cause systematic bias in the other model parameters, particularly the radius.

Finally, this study was conducted at 1.5 T with gradient separations corresponding to diffusion times ranging from 17.7 to 51.7 ms. Higher-field scanners often have stronger gradients, which would allow shorter diffusion times to be examined. A wider range of scan parameters, along with protocol optimization techniques (22), would allow design of protocols that are more sensitive to restriction effects and may also allow relaxation of the constraints used to make fitting tractable.

In conclusion, this study showed that microstructure methods capture additional information at high *b*-values at 1.5 T in bone metastases. The kurtosis, intracellular volume fraction, and radius parameters from kurtosis and VERDICT fitting are not simply related to the ADC and may yield new information. Obtaining histological samples from bone is very challenging and, therefore, non-invasive methods of inferring tissue characteristics are needed for treatment monitoring. The usefulness of high *b*-value, multi-diffusion time protocols, and more complex models should be further explored for characterizing response to therapy in metastatic bone disease.

## Ethics Statement

This study was carried out in accordance with the recommendations of HIPAA (Health Insurance Portability and Accountability Act) guidelines and the local Research Ethics Committee with written informed consent from all subjects. All subjects gave written informed consent in accordance with the Declaration of Helsinki. The protocol was approved by the local Research Ethics Committee (Royal Marsden NHS Foundation Trust).

## Author Contributions

Study design: CB, EP, and DC. Pulse sequence design: TF. Data acquisition: VM, NT, MO, and DC. Data analysis and interpretation: CB, NT, MO, DC, and EP. Project management: EP, DA, DH, and ML. Manuscript preparation: All.

## Conflict of Interest Statement

TF is an employee of Siemens Healthcare. The other authors declare that the research was conducted in the absence of any commercial or financial relationships that could be construed as a potential conflict of interest.
