# Supplementary material for: Microstructure Characterization of Bone Metastases from Prostate Cancer with Diffusion MRI: Preliminary Findings
Source: Front Oncol. 2018 Feb 16;8:26. doi: 10.3389/fonc.2018.00026 (PMC5820304; doi:10.3389/fonc.2018.00026)
Supplement: Supplementary file 1 [file Presentation_1.PDF]

Supplementary Material

Microstructure Characterization of Bone Metastases from Prostate Cancer with Diffusion MRI: Preliminary Findings

Colleen Bailey*, David J Collins, Nina Tunariu, Matthew R Orton, Veronica A Morgan, Thorsten Feiweier, David J Hawkes, Martin O Leach, Daniel C Alexander and Eleftheria Panagiotaki

*** Correspondence:** Corresponding Author: colleen.bailey@sunnybrook.ca

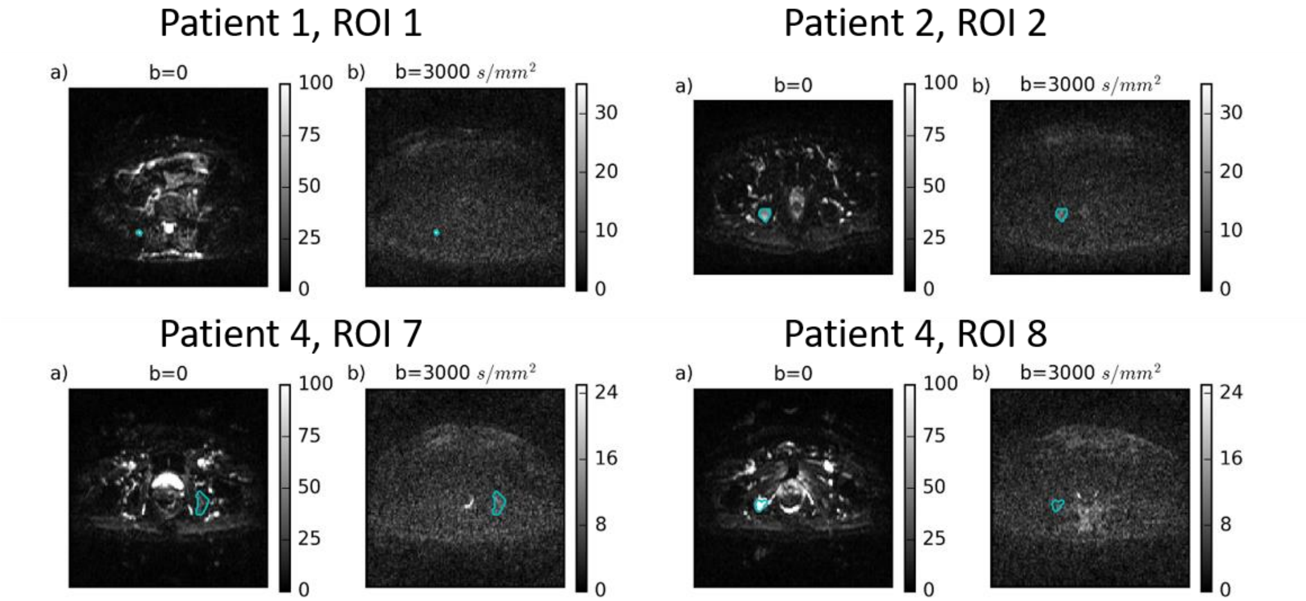

Supplementary Figure 1. Regions of interest on b=0 and b=3000 s/mm^2 diffusion-weighted images for the remaining four ROIs not shown in Figure 1.

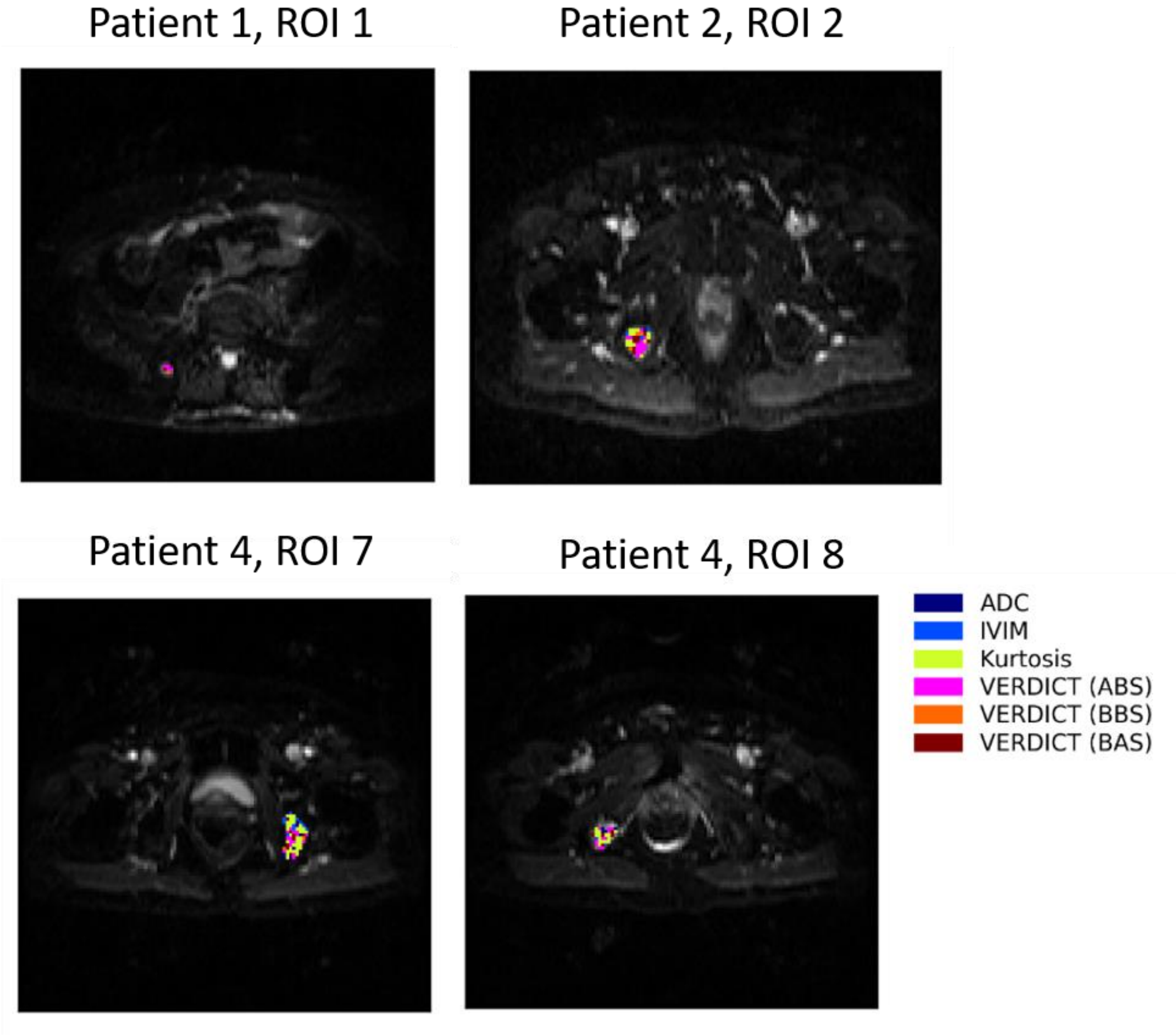

Supplementary Figure 2. Model selection results for the remaining ROIs not shown in Figure 2a. The maps show the model that best explains the data (lowest AIC) in each voxel.

Patient 1, ROI 1

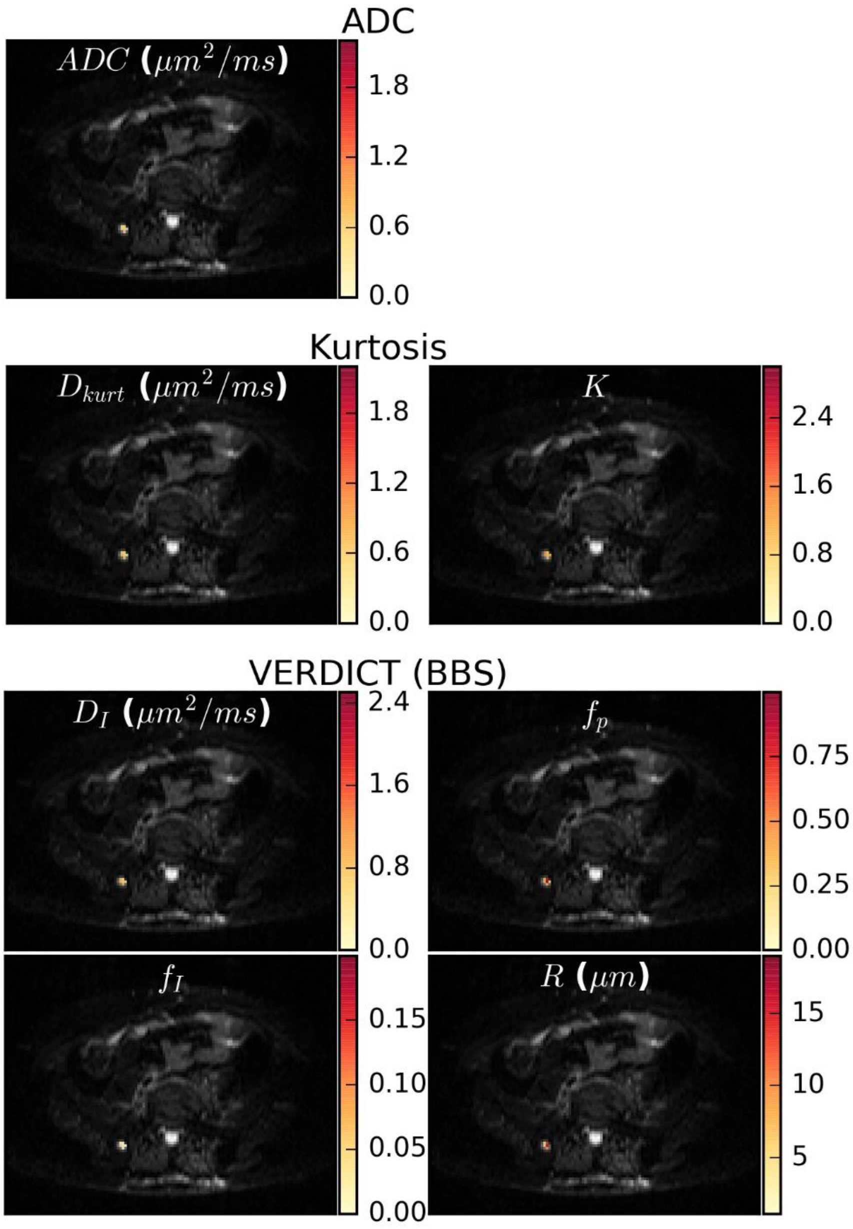

Patient 2, ROI 2

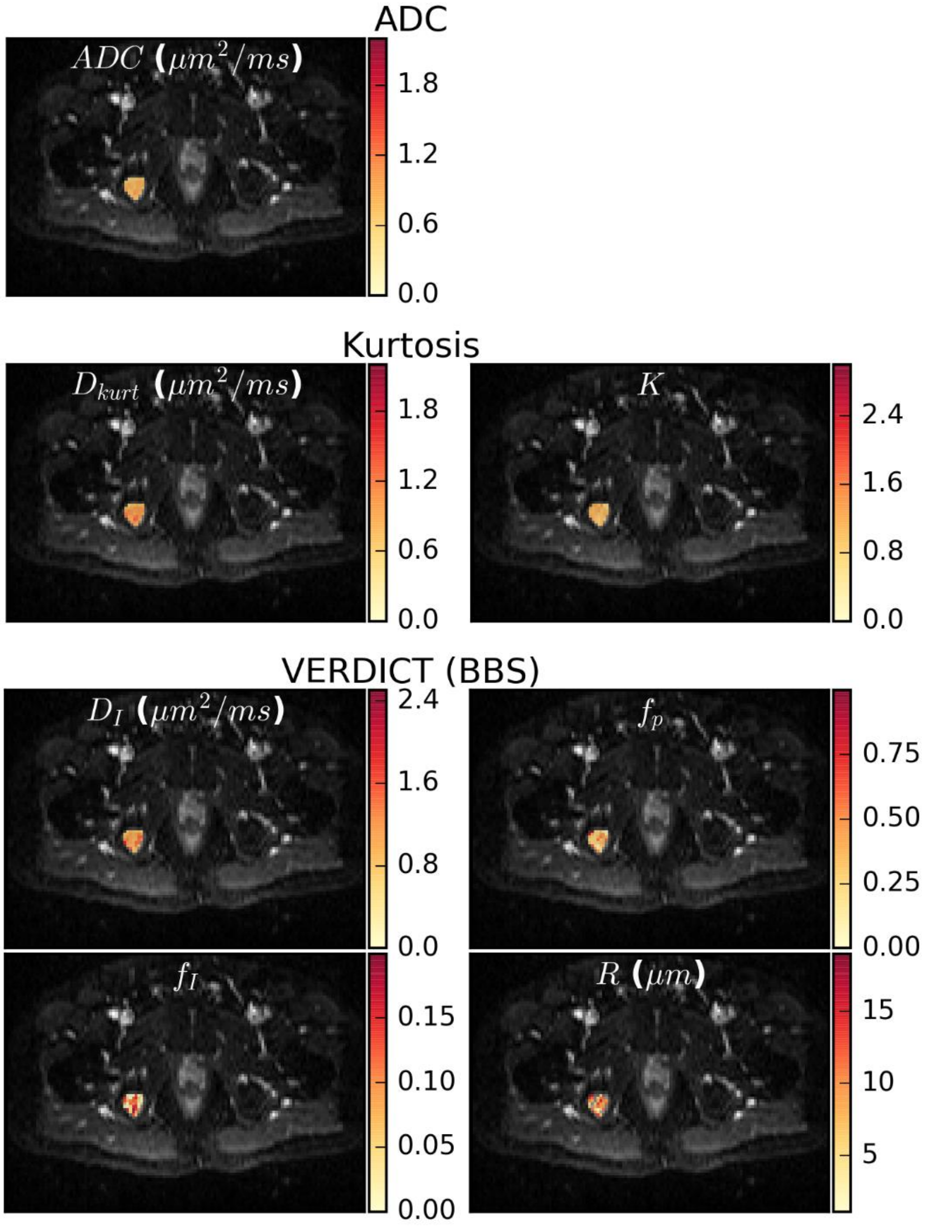

Patient 4, ROI 7

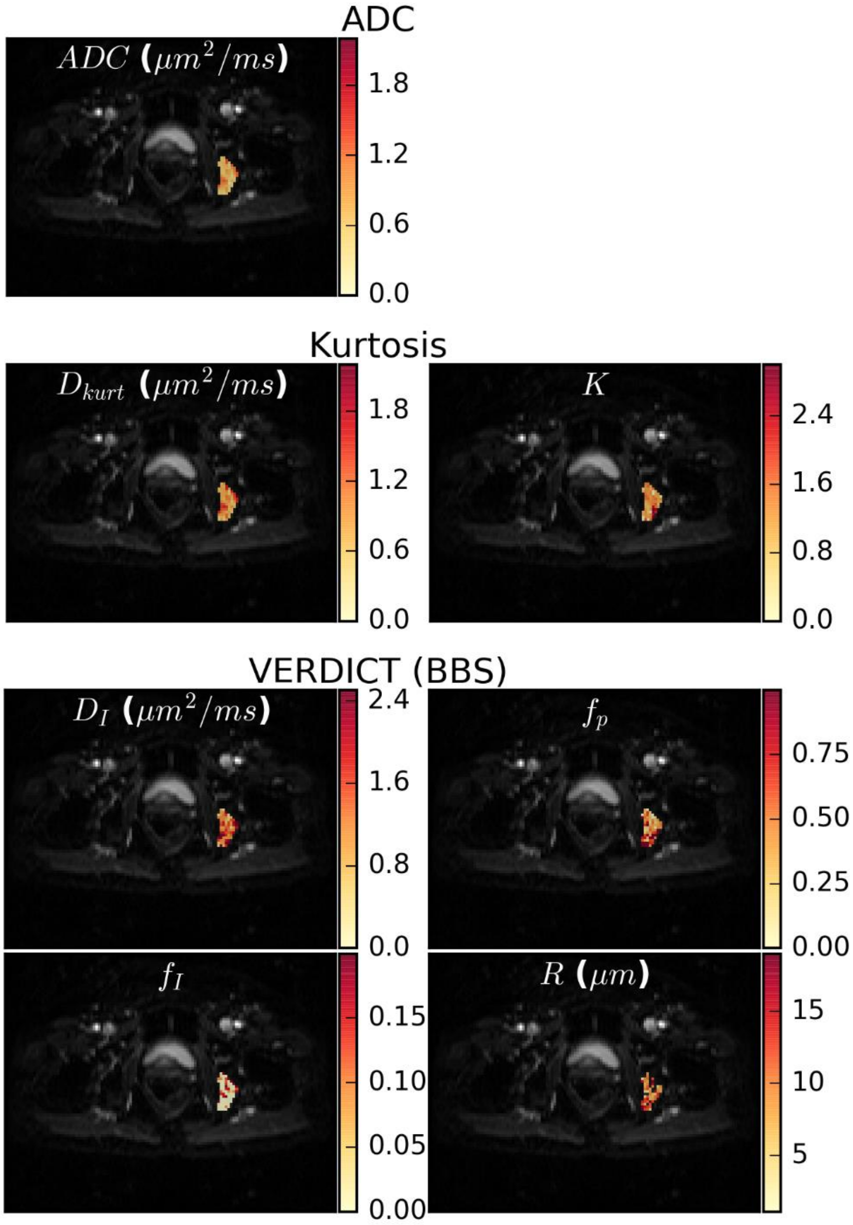

Patient 4, ROI 8

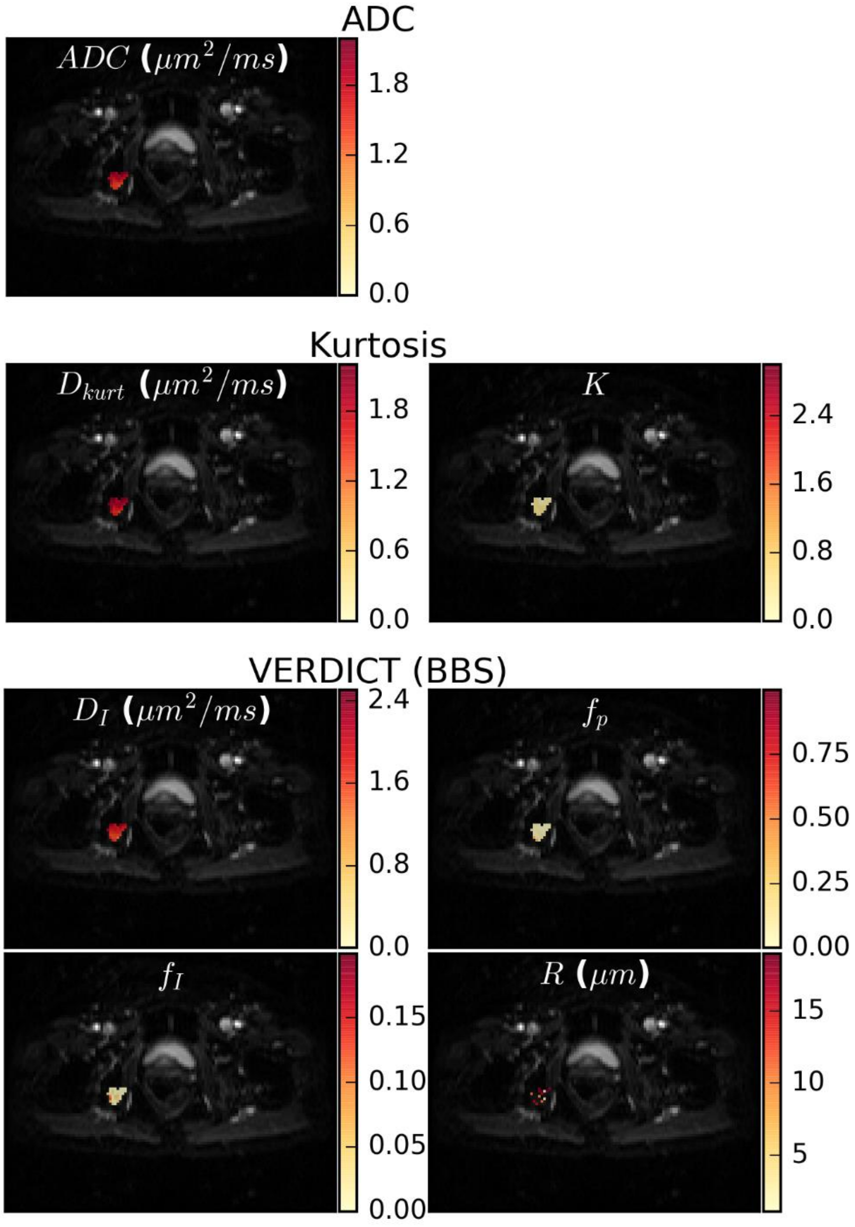

Supplementary Figure 3. Model parameter maps for the four ROIs not shown in Figure 4.
